# Unprecedented extreme depletion of Earth's ionosphere during the May 2024 super geomagnetic storm revealed by the Chinese Meridian Project

**DOI:** 10.1093/nsr/nwaf369

**Published:** 2025-09-08

**Authors:** Jinbin Cao

**Affiliations:** School of Space and Earth Sciences, Beihang University, China

The terrestrial ionosphere is a highly complex geospace environment that exhibits not only significant climatological changes but also pronounced space weather variations caused by solar and geomagnetic disturbances. These ionospheric variations can greatly impact radio wave propagation, adversely affecting the operations of modern technological systems such as shortwave communication, satellite navigation and positioning services. During intense geomagnetic storms, ionospheric behavior becomes particularly complex and difficult to predict due to substantial energy and momentum injections, as well as various electrodynamic and dynamic processes. Consequently, accurately characterizing storm-time ionospheric disturbances and uncovering their underlying mechanisms, especially during super-geomagnetic storms, remains a critical yet challenging task for the space weather research community.

The super-geomagnetic storm of 10–12 May 2024, with a minimum disturbance storm time (Dst) index of −412 nT, was the most intense storm in the past two decades. It provides an unprecedented opportunity to investigate global ionospheric behavior under extreme forcing. Recently, using data from the Chinese Meridian Project (CMP) monitoring network [1] and other multi-instrument ground- and space-based measurements, Chen *et al.* [2] reported unprecedented observations of extreme ionospheric electron density depletion and its hemispheric asymmetry during this storm, addressing a key gap in space weather research. The study revealed a dramatic storm-time depletion of ionospheric electron density, with a maximum reduction of 98% over China and across the entire Northern Hemisphere for more than 2 days. The largest total electron content (TEC) depletion reached 100 TEC units in low-latitude regions over the East Asian sector, accompanied by the suppression and eventual disappearance of the northern crest of the equatorial ionization anomaly (EIA). Furthermore, this extensive depletion led to a complete loss of ionospheric backscatter echoes in multiple ionosondes within the CMP network for an extended period. Analysis of vertical plasma drift and ΣO/N_2_ ratios indicated that the extreme depletion was primarily driven by neutral composition disturbances propagating from high to low latitudes, combined with the effects of a westward electric field caused by overshielding penetration and disturbance dynamo electric fields at low latitudes.

In addition to extreme depletion in the Northern Hemisphere, Chen *et al.* [2] also observed significant hemispheric asymmetry in the global ionosphere. The mid- to low-latitude ionosphere in the Southern Hemisphere exhibited considerable electron density enhancements, showing a striking contrast to the Northern Hemisphere's depletion. By combining multi-instrument observations with Thermosphere–Ionosphere Electrodynamics General Circulation Model (TIEGCM) simulations, the team demonstrated that this hemispheric asymmetry was driven by summer-to-winter neutral winds and differences in ΣO/N_2_ ratios between the two hemispheres.

This study documents the most extreme ionospheric depletion ever recorded, driven by a super-geomagnetic storm. The hemispheric asymmetry—depletion in the north and enhancement in the south—challenges existing storm models. It establishes definitive links between magnetospheric energy injection, thermospheric composition changes and global ionospheric restructuring. These observations provide critical benchmarks for space weather model validation, particularly regarding extreme-event radio blackout prediction. Future work will quantify latitudinal dependencies of depletion thresholds and refine asymmetry parametrizations in coupled magnetosphere–ionosphere models.
